# *LIN* *28B* expression is downregulated in mature spermatozoa of oligozoospermic men and associates with genetic variants previously linked to pubertal onset

**DOI:** 10.1530/EC-25-0044

**Published:** 2025-05-28

**Authors:** María Cecilia Lardone, Marina Díaz-Fontdevila, Eliana Ortiz, Germán Iñiguez, Pamela Inostroza, Cristóbal Espinoza, Mauricio Ebensperger, Kristian Almstrup, Andrea Castro

**Affiliations:** ^1^ Institute of Maternal and Child Research, School of Medicine, University of Chile, Santiago, Chile; ^2^ Centro de Investigación Clínica Aplicada (CICA), School of Medicine, University of Chile, Santiago, Chile; ^3^ Urology Department, San Borja Arriarán Clinical Hospital, Santiago, Chile; ^4^ Department of Growth and Reproduction, Rigshospitalet, University of Copenhagen, Copenhagen, Denmark; ^5^ Department of Cellular and Molecular Medicine, University of Copenhagen, Copenhagen, Denmark

**Keywords:** LIN28B, miRNA let-7, oligozoospermia, LIN28B genetic polymorphisms, male infertility, male puberty

## Abstract

LIN28B is an RNA-binding protein that acts as a post-transcriptional regulator of genes involved in developmental timing and self-renewal through its interaction with *let-7* miRNAs. Large-scale genomic studies have strongly implicated SNPs in *LIN28B* with male puberty timing. In addition, the occurrence of late puberty is linked to diminished semen quality in adult life. Therefore, we aimed to study the association of puberty-linked *LIN28B* genetic variants with semen parameters, reproductive hormones and the spermatozoa expression of the *LIN28B/Let-7 axis* in idiopathic oligozoospermic men. One hundred and eleven oligozoospermic (cases) and 258 men with normal sperm concentration (controls) were genotyped for five *LIN28B* SNPs (rs7759938, rs395962, rs314268, rs314277 and rs314280). The abundance of the *LIN28B* transcript, *let-7a* and *let-7c* miRNAs were measured by qRT-PCR in RNA isolated from purified sperm. Serum blood samples were analysed for reproductive hormones. Lower abundance of the *LIN28B* transcript and higher expression of *let-7c* were observed in cases (*P* < 0.001). Furthermore, rs395962_T was associated with a reduced abundance of the *LIN28B* transcript in cases (dominant: *P* = 0.032). On the other side, we observed a positive association of rs314277_A (Additive: *P* = 0.024), rs7759938_C (dominant: *P* = 0.025) and rs314280_A (dominant: *P* = 0.024) with total testosterone levels in cases. The decreased transcript abundance of *LIN28B* in sperm of idiopathic oligozoospermic men and its association with SNPs known to affect the onset of puberty and total testosterone levels suggests a role for LIN28B in the primary impairment of spermatogenesis through its expression in early germ cells or regulating the testicular biosynthesis of testosterone at a central level. Therefore, our results provide a potential mechanistic link between the regulation of pubertal timing and adult testicular function.

## Introduction

Spermatogenesis is a complex process that takes place in the testis and involves the proliferation and differentiation of spermatogonia into mature spermatozoa. In humans, spermatogenesis is initiated around 8 weeks after birth when spermatogonial stem cells differentiate into spermatogonia (1). However, complete spermatogenesis only occurs after the onset of puberty when the differentiated spermatogonia are allowed to enter meiosis and subsequently go through spermiogenesis. Interestingly, studies have shown that a self-reported late onset of puberty is related to reduced sperm concentration, total sperm counts and testosterone levels in young healthy adults, suggesting that the age of pubertal onset may be associated with male reproductive function later in life (2, 3, 4). The age of pubertal onset shows a high degree of variability within the general population, and the cumulative effects of common genetic variants such as single nucleotide polymorphisms (SNPs) have been shown to contribute to this variability (5). In this regard, various large-scale genomic studies have revealed that single SNPs in or near the *LIN28B* gene represent the most significant common genetic variants associated with the onset of puberty in males. These SNPs are primarily responsible for delaying puberty, as evidenced by a later age at voice break (rs395962_T) (5, 6) and Tanner genital stage in boys (rs314268_G) (7). In addition, considering the strong genetic correlation between the timing of puberty in males and females (8), it is plausible to suggest that other *LIN28B* genetic variants linked to delayed menarche, including rs7759938_C (9, 10), rs314280_T (11) and rs314277_A (11), may also be implicated.

*LIN28B* encodes an RNA-binding protein that functions as a post-transcriptional regulator of genes involved in developmental timing and self-renewal in embryonic stem cells (12, 13). In adult animals, *LIN28B* expression is restricted to the testis, placenta and hypothalamus (14, 15, 16). Similarly, in adult humans, *LIN28B* is mainly expressed in the testis and placenta (https://gtexportal.org/home/gene/LIN28B), and within the adult human testis, *LIN28B* transcripts are found in the germ cells and not in the somatic cells (17, 18, 19). Thus, the relatively restricted expression of *LIN28B* in the adult human testis and its genetic association with pubertal onset suggests a potential regulatory link to gonadotropin drive (14), and proposes a putative role for LIN28B in testicular function.

It is well-established that LIN28B acts as an RNA-binding protein inhibiting the biogenesis of the let-7 family of microRNAs, which are critical for many cellular functions (20, 21). Consistent with this regulatory role, *Lin28b* has been described to show an inverse pattern of expression to *let-7* in the rodent testis (16). However, limited information is available on the regulation of the LIN28/let-7 axis in human testicular tissue and even more so in cases of spermatogenic impairment.

The causes of impaired spermatogenesis are largely unknown in most infertile men. The most current reports estimate that 40–60% of infertile patients with spermatogenic impairment have an unexplained cause of infertility, and among individuals with oligozoospermia, the percentage of unexplained cases approaches 80% (22).

With this background, we speculate that since LIN28B is associated with pubertal development, it would also have a role in the maturation of the spermatogenic and steroidogenic functions of the adult testis. Therefore, our research aimed to investigate whether a dysregulation in the expression of the *LIN28B/Let-7* axis in sperm cells accounts for impaired seminal quality in men with secretory oligozoospermia. Due to the practically null transcriptional activity of spermatozoa, we speculate that *LIN28B* transcript abundance in sperm originates from expression in immature germ cells. In addition, we sought to examine the association of *LIN28B* SNPs, known to affect pubertal onset, with reproductive hormones and semen parameters in oligozoospermic and normozoospermic men.

## Materials and methods

### Study population

This study was approved by the Research Ethics Committee on Human Beings, School of Medicine, University of Chile (approval #101/2020). Subjects were recruited from men who consulted for diagnosis of male or partner infertility at the Institute of Maternal and Child Research, University of Chile, between June 2021 and January 2024. Male patients aged 18–55 years with an indication for sperm analysis without a history of azoospermia or obstructive oligozoospermia were invited to participate. Participants signed an informed consent at the time of recruitment and acceptance of sample collection. The patients were thoroughly interviewed to obtain a complete general, urological and andrological history to exclude patients with previous or current events of infection and/or inflammation of the reproductive tract, excessive consumption of tobacco, alcohol and drugs, exposure to hormonal therapy, bariatric surgery, testicular cancer as well as chronic diseases. A urologist performed the physical and urological evaluation aimed at detecting varicocele, palpation of the epididymis and vas deferens, evaluation of androgenisation, and measurement of testicular volume using an orchidometer. In addition, participants were asked regarding the age of onset of puberty-related changes, explicitly recalling the appearance of pubic hair, voice break or penile growth. Further, they were asked to indicate whether these changes occurred at the same time, earlier or later compared with their peers.

Karyotyping and detection of Y-chromosome microdeletions (YMD) were performed in patients with sperm concentration <5 × 10^6^/mL. Karyotyping was performed using the G-banding method, counting 35 mitoses in leukocytes, and YMD was performed on DNA extracted from peripheral blood, as reported earlier (23). Blood samples were obtained between 08:00 and 11:00 h for serum hormone analysis. LH and FSH levels were measured by immunoradiometric assay (Izotop, Hungary), and total testosterone was measured by radioimmunoassay (DIAsource Immunoassays S.A, Belgium). Patients were excluded if they had an abnormal karyotype, Y-chromosome microdeletions, the presence of grade III and IV varicocele, hypogonadotropic hypogonadism, seminal infection, epididymitis, scrotal trauma or injury, or evidence of previous scrotal or inguinal surgeries.

### Semen analysis

Semen analysis was performed following the guidelines of the Laboratory Manual for the Examination and Processing of Human Semen, WHO, 5th ed. (WHO5) (24) on a seminal sample obtained by masturbation with sexual abstinence between 2 and 7 days. Briefly, semen samples were allowed to liquefy for at least 30 min before the analysis was initiated. Sperm concentration was assessed using a Neubauer hemocytometer, counting two times within the acceptable variations and at least 200 spermatozoa per replicate. Sperm motility was classified as progressive, non-progressive or immotile during the evaluation of a wet preparation and counting at least 200 sperm per duplicate. Strict criteria were used to evaluate human sperm morphology using Spermac Stain reagent (FertiPro NV, Belgium) in at least 200 spermatozoa under high magnification (1,000×).

### Quantification of *LIN28B* transcript in spermatozoa

#### Purification of spermatozoa

The isolation of spermatozoa from the semen sample was carried out through a 40% density gradient. Briefly, 1 mL of PureSperm 40® (Nidacon, Sweden) and up to 1.5 mL of semen were added to a 15 mL tube and centrifuged at 300 ***g*** for 20 min. The resulting pellet was transferred to a new tube, and two centrifugations were performed with 5 and 1 mL of PBS 1× at 300 ***g*** for 15 min each. The number of sperm and round cells were counted both before and after purification, and the mean sperm recovery rate, as well as the percentage of round cell removal after semen processing on the density gradient, were 76% ± 23 and 87% ± 16%, respectively.

#### Sperm RNA extraction

Total RNA was extracted from separated sperm cells using the combined method of separation with guanidinium thiocyanate-phenol-chloroform (Trizol®, Invitrogen, USA) and purification by affinity column (RNeasy Mini Kit, Qiagen, Germany) according to previous publications (25), with some modifications. After separation, sperm cells were lysed with 0.5 mL of RLT buffer (RNeasy Mini kit, Qiagen) added with 7.5 μL of β-mercaptoethanol using the homogenizer (Pro Scientific 200). Next, a second homogenization was carried out using 500 μL of Trizol, and then 200 μL of chloroform was added to the homogenate. The phases were separated after vigorous shaking and centrifugation at 12,000 ***g*** for 20 min at 4°C. An equal volume of 100% ethanol was added to the resulting aqueous phase to purify the sample, and the separation of the RNA was continued on the affinity column according to the manufacturer’s instructions (RNeasy Mini Kit, Qiagen). The RNA was treated with RNase-Free DNase (Qiagen) in the same column to eliminate DNA contamination, following the manufacturer’s instructions. RNA was recovered by eluting with 20 μL of nuclease-free water heated to 65°C. Finally, the RNA was quantified by spectrophotometry (NanoDrop™, USA), and the aliquoted RNA was stored at −80°C until use.

#### Real-time PCR (qPCR)

The abundance of the *LIN28B* transcript in sperm RNA was quantified by real-time PCR with SYBR Green I on the StepOne Plus instrument (Applied Biosystems, USA). Initially, cDNA was synthesized from total RNA using Revert Aid H minus reverse transcriptase (Thermo Scientific, USA) and oligo d(T) 12–18 (Invitrogen) following the manufacturer’s instructions. Aliquots of 500 ng of total RNA were used for cDNA synthesis. The qPCR reactions were performed using SYBR® MasterMix dTTP Blue (Takyon, Belgium) and primers specially designed for specific amplification of *LIN28B* (NM_001004317.4) and *GAPDH-S* (NM_014364.5) transcripts (PrimerBlast tool): *LIN28B*-forward, AGC​ACA​TTA​GAC​CAT​GCG​AGC and *LIN28B*-reverse, TTTGCTAGCCGCCTTCG (fragment size 156 bp) and *GADPHS*-forward, 5′-CAT​GAA​CAT​TGT​GAG​CAA​CGC-3′ and *GADPH-S*-reverse, 5′-CAA​CCC​TTC​CAC​GAT​CCC​AA-3´ (fragment size 94 bp). The expression of the *LIN28B* mRNA was normalized to the expression of the housekeeping gene *GAPDH-S* (sperm-specific glyceraldehyde-3-phosphate dehydrogenase) using the 2^ΔCT^ method. All samples were measured in triplicate, and replicates that generated a standard deviation greater than 0.5 were eliminated. The efficiencies of the qPCR amplification reactions for the *LIN28B* and *GAPDH-S* transcripts were determined by amplifying five serial dilutions of cDNA (125 ng/μL to 7.81 ng/μL) obtained from total RNA of testis with conserved spermatogenesis. The efficiencies of the amplification reactions were 101 and 87%, with *R* ^2^ = 0.983 and 0.93 for the amplification of *LIN28B* and *GAPDH-S* transcripts, respectively.

To evaluate the expression of *LIN28B* and *GAPDH-S* mRNA in germ cells, total RNA obtained from human testicular tissues and white blood cells were subjected to RT-PCR analysis, employing the same conditions and primers mentioned above (Supplementary Materials and methods (see section on Supplementary materials given at the end of the article)).

### Quantification of *let-7* miRNAs in spermatozoa

The abundance of *hsa-let-7a-5p* and *hsa-let-7c-5p*, as representative members of the *let-7* microRNA family, was quantified by qRT-PCR. *Hsa-miR-30a-5p* was selected as endogenous expression controls based on a previous study (26). A 50 ng aliquot of total RNA measured by fluorimetry (Qubit™ RNA HS Assay Kit) was reverse transcribed using TaqMan® Advanced MicroRNA cDNA Synthesis Kit (Applied Biosystems), following the supplier’s instructions (Supplementary Fig. 1). The qPCR reaction was carried out on the StepOne Plus Real-Time PCR equipment (Applied Biosystems) using TaqMan Universal Master Mix II, no UNG (Applied Biosystems), and previously designed primers and probes (TaqMan® Advanced miRNA Assays, Applied Biosystems). Each sample was measured in triplicate, and replicates whose standard deviation exceeded 0.5 from the average were eliminated.

### Single nucleotide polymorphism selection and genotyping

SNPs in *LIN28B* were selected based on their reported association with pubertal traits, such as delayed age at menarche (rs7759938_C (9, 10), rs314280_T (11), rs314277_A (11), rs395962_T (5, 27, 28)), delayed age at voice break in boys (rs395962_T) (5, 6), Tanner genital stage in boys (rs314268_G) (7, 29), and taking into consideration the high genetic correlation between male and female puberty timing (8). Selected SNPs are located within a single block of approximately 30 Kb (GRCh38) in high linkage disequilibrium (LD), mean *D´* = 0.99 (Supplementary Fig. 2). Among these *LIN28B* SNPs, rs7759938 is located upstream of the *LIN28B* gene, while rs314280 is in a regulatory region of the *LIN28B*, and rs314268, rs314277 and rs395962 are intronic variants (Ensemble regulation release 113 – October 2024).

Genomic DNA was extracted from peripheral blood using the Wizard DNA purification kit (Promega). DNA quantification was performed by spectrophotometry (NanoDrop™) and stored at −20°C until use. The five SNPs were genotyped using the TaqMan™ SNP Genotyping Assay (Thermo Fisher Scientific) (Assay ID: C_11906296_20, C_629975_20, C_629964_10, C_629970_20 and C_1361832_10) in a StepOne Plus thermocycler with 2× TaqMan® Genotyping Master Mix (Applied Biosystems). Raw data from genotyping experiments were analysed with TaqMan® Genotyper™ Software. All the SNPs have a minor allele frequency >0.05, according to the 1000 Genomes Project Phase 3 (in European and American populations).

### eQTL analysis of public access data

The GTeX database (https://gtexportal.org/home/gene/LIN28B) were examined to compute, on demand, the association between the five SNPs (rs7759938, rs314268, rs314277, rs314280 and rs395962) and the abundance of *LIN28B* transcript in testicular tissue, using the tool eQTL Calculator in the current release of GTEx Analysis V8.

### Statistical analysis

The estimation of the statistical power of this study was performed using the GAS power calculator for one-stage genetic association studies available at http://csg.sph.umich.edu/abecasis/cats/gas_power_calculator/, University of Michigan, School of Public Health, Department of Biostatistics Center for Statistical Genetics. Deviances from Hardy-Weinberg equilibrium (HWE) were evaluated in the control group at the 0.05 significance level using the function *tableHWE* test of the ‘SNPassoc’ package in R. Genetic association between SNPs and the case–control design or reproductive parameters (FSH, LH, testosterone, inhibin B, sperm concentration and sperm count) was assessed using a general lineal model under four different comparative models (codominant, dominant, recessive and additive) using the R package ‘SNPassoc’. The analysis was adjusted for confounders as stated in each case. Continuous variables that deviate from normal distribution were transformed to square root (sperm concentration and total sperm count) or natural logarithmic transformation (FSH, LH, testosterone levels, testosterone/LH and inhibin B/FSH ratios). The percentage change was calculated using the formula (10^^β^^−1)×100%. The software package SPSS version 21 was employed for statistical analysis of differences between means and correlation between hormone levels, seminal parameters and gene expression levels using non-parametric tests (Mann–Whitney U test and Kruskal–Wallis test), and Spearman’s correlation coefficient, respectively. Statistical significance was considered with a *P* value < 0.05.

## Results

### Subjects

A total of 369 men attending our clinic for andrological evaluation were enrolled in this study. Based on semen analysis, 111 men were classified as oligozoospermic cases (presence of spermatozoa in the ejaculate <15 × 10^6^/mL, cases) and 258 were included as normozoospermic controls (sperm concentration ≥15 ×10^6^ spermatozoa/ml) following the World Health Organization guidance and reference limits for semen analysis (24). No azoospermic or cryptozoospermic patients were included. Semen parameters and hormonal profiles of both groups are shown in Table 1. As expected, sperm concentrations and total sperm count were significantly lower in the cases compared with the controls. In addition, cases had significantly fewer progressive motile sperm, fewer morphologically normal sperm and a lower vitality compared with controls. Among the cases, 36% could be further sub-classified as asthenozoospermic (<32% progressive motility), while only 9.8% of the controls had decreased motility. As expected from the semen parameters, the cases showed significantly higher serum concentrations of FSH and LH, a lower testosterone/LH ratio, but with only a slight increase in the serum concentration of total testosterone, suggesting a secretory origin of the low sperm count. Age was similar between cases (35.3 ± 5.9, range: 24–51 years) and controls (35.5 ± 5.8, range: 20–52 years) (*P* = 0.811). In addition, no significant difference was observed in the mean body mass index (BMI) between both groups (28.8 ± 4.8, range: 18.9–42.3 vs 28.7 ± 4.8, range: 18.4–48.9) (*P* = 0.835).

**Table 1:** Seminal and hormonal features in cases and controls.

	Cases	Controls	*P* value
*n*	111	258	
Age	35.3 ± 5.9 (20–52)	35.5 ± 5.7 (22–52)	0.811
BMI	28.8 ± 4.8 (18.9–42.3)	27.6 ± 3.4 (18.8–48.9)	0.835
Semen volume (mL)	2.6 (0.8–6)	2.6 (1–5.4)	0.303
Sperm concentration (million/mL)	3.3 (0.1–13.9)	57 (18.2–188)	**<0.001**
Total sperm count (million)	8.4 (0.4–47.6)	158 (35.2–568)	**<0.001**
Progressive motility (%)	46 (1–75)	64 (22–81)	**<0.001**
Asthenozoospermia (%)	36	9.8	**<0.001**
Normal morphology (%)	1 (0–4)	2 (0–6)	**<0.001**
Vitality (%)	81 (36–93)	88 (73–95)	**<0.001**
FSH (mIU/mL)	5.6 (1.6–20.7)	3.1 (1.4–7.4)	**<0.001**
LH (mIU/mL)	4.3 (1.4–10.1)	3.1 (1.3–5.6)	**<0.001**
Testosterone (nmol/L)	11.4 (6.9–19.1)	10.4 (5.6–17)	**0.012**
T/LH ratio	2.7 (1.2–7.1)	3.6 (1.6–7.8)	**0.002**

Values are expressed as mean ± SD (range) or median (5th–95th percentile). Statistically significant *P* values are shown in bold letters. Reference values: FSH: 1–7 mIU/mL; LH: 1–8 mIU/mL; testosterone (T): 6.9–27.7 nmol/L; BMI, body mass index.

When patients were asked to recall the timing of their puberty, 87% of the recruited patients were able to answer the age or relative age of the appearance of pubertal changes (i.e. appearance of pubic hair, voice break or penile growth). Based on the self-report, 5.6, 69 and 25% of the cases, and 6.7, 78.2 and 15.1% of the controls stated having experienced early, concurrent or delayed puberty in comparison to their peers, respectively. When cases and controls jointly were grouped based on their self-reported relative age of puberty, we observed that patients who reported a delayed age of puberty had a significantly lower sperm concentration, lower total sperm count, decreased progressive motility and lower percentage of normal sperm morphology compared with patients reporting synchronous or early onset of puberty (Supplementary Table 1). Furthermore, multiple linear regression analysis corroborated a tendency of a decreased sperm concentration by 23% (95% CI −41.5; 1.7), a decreased total sperm count by 25% (95% CI −44.3; 0.9), and an increased FSH level by 12% (95% CI −0.9; 27.2) in men with late onset of puberty compared with synchronous onset (Supplementary Table 2).

### Expression of *LIN28B/let 7* in spermatozoa of cases and controls

**Figure 1:**
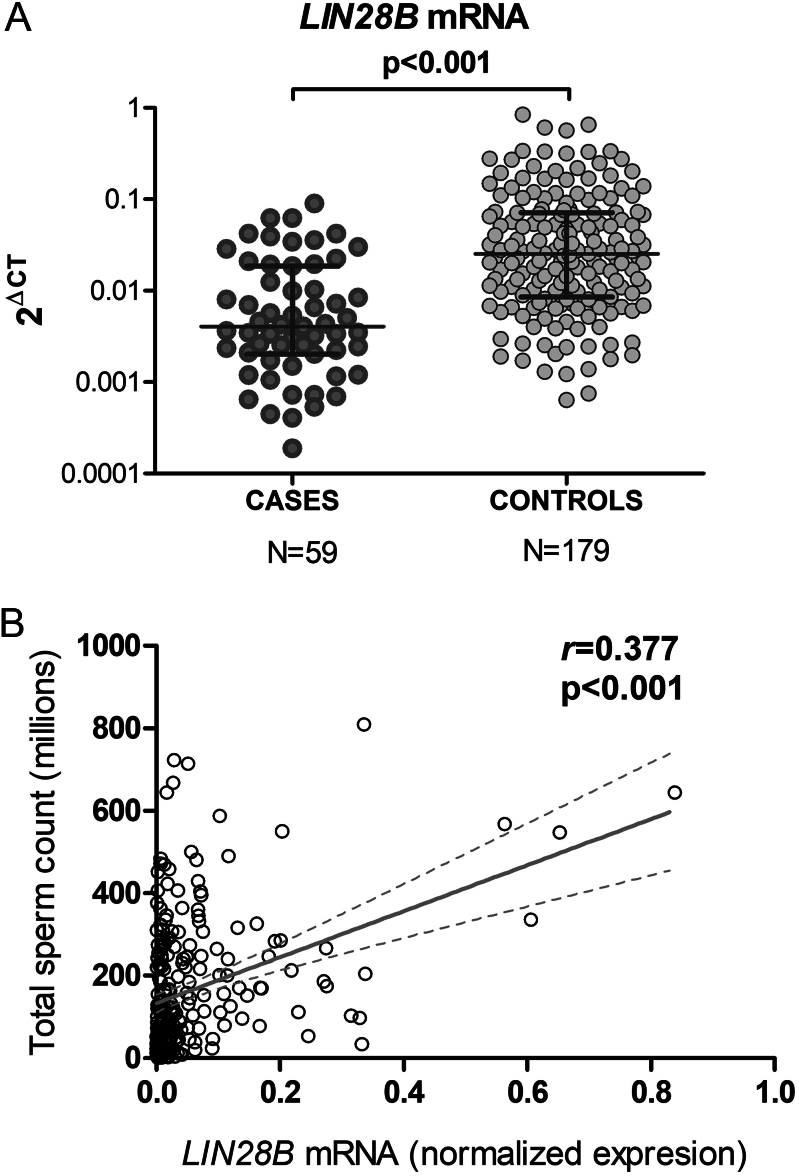
Relative quantification of LIN28B transcript abundance in sperm cells of subjects with oligozoospermia (cases) and subjects with normal sperm count (controls). (A) LIN28B mRNA expression is normalized to the reference gene GAPDH-S using the 2ΔCt method. The lines show the median, 25th and 75th percentile. *P < 0.001, Mann–Whitney test. (B) Correlation and regression analysis between LIN28B normalized expression and total sperm count in cases and controls (*n* = 238). The line shows the regression line and the 95% confidence band.

To investigate the transcriptional expression of *LIN28B* in male germ cells, we measured the relative abundance of *LIN28B* mRNA in sperm samples collected from 238 participants, comprising 59 cases and 179 controls, from which enough RNA was successfully extracted for the subsequent RT-PCR analysis. The comparison between the groups revealed a significantly decreased abundance of the *LIN28B* transcript in sperm from cases compared with controls (*P* < 0.001) (Fig. 1A). Further, regression analysis showed a significant correlation between normalized *LIN28B* mRNA abundance and sperm concentration and total sperm count (*r* = 0.329, *β* = 0.319, *R* ^2^ = 0.108, *P* < 0.001 and *r* = 0.377, *β* = 0.372, *R* ^2^ = 0.142, *P* < 0.001, adjusted by age and BMI), explaining a 14% in the total sperm count variability (Fig. 1B).

The expression of *LIN28B* and *GAPDH-S* transcripts was exclusively observed in spermatozoa and testicular tissues with germ cells. DNA samples and mRNA from peripheral blood leukocytes did not amplify with the mentioned primers (Supplementary Figs 3 and 4), confirming that the abundance of the quantified transcripts in our sperm-purified samples represents the expression of *LIN28B* per ejaculated spermatozoa.

**Figure 2:**
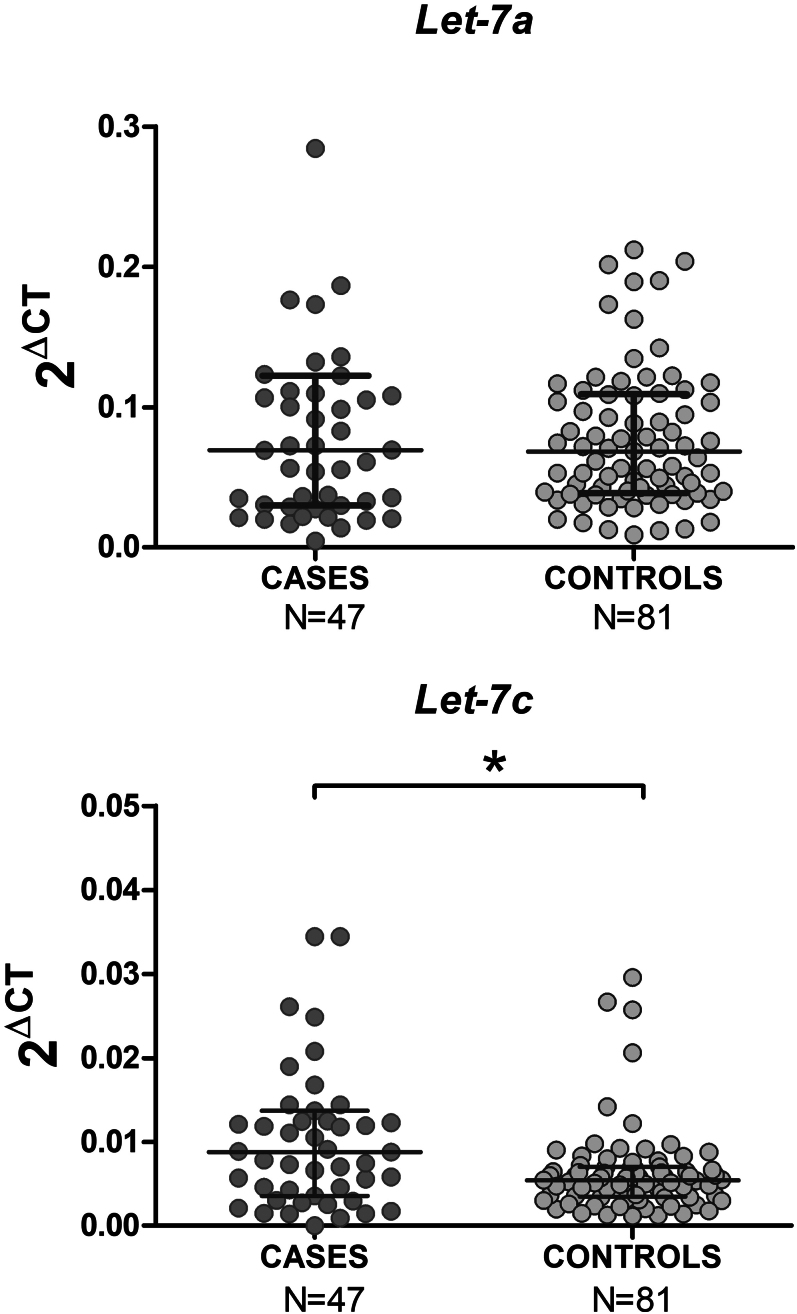
Relative quantification of hsa-let-7a-5p and hsa-let-7c-5p miRNAs abundance in sperm cells of subjects with oligozoospermia (cases) and subjects with normal sperm count (controls). hsa-let-7a-5p and hsa-let-7c-5p expression is normalized to the miRNA hsa-30a-5p using the 2ΔCt method. The lines show the median, 5th and 95th percentile. *P < 0.05, Mann–Whitney test.

The abundance of *hsa-let-7a-5p* and *hsa-let-7c-5p* was measured in spermatozoa of 47 cases and 81 controls. The normalized abundance of *hsa-let-7a-5p* and *hsa-let-7c-5p* to the abundance of *hsa-miR-30a-5p* revealed a significant overexpression of *hsa-let-7c-5p* in cases compared with controls (*P* = 0.004) (Fig. 2), while no difference was detected for *hsa-Let-7a-5p* (*P* = 0.931).

In agreement with the proposed role of LIN28B as a negative regulator of the biogenesis of *let-7* family, a significant inverse correlation was found between the expression levels of *LIN28B* and *hsa-Let-7c-5p* (*r* = −0.230; *P* = 0.010) (Supplementary Fig. 5).

The differences observed in the expression levels of *LIN28B* and *hsa-Let-7c-5p* remained statistically significant after grouping the cases according to total sperm count (*P* < 0.001 and *P* = 0.003, respectively). In addition, *hsa-let-7c-5p* abundance correlated inversely with sperm concentration and total sperm count parameters (*r* = −0.281, *P* = 0.001 and *r* = −0.305, *P* < 0.001).

### Association of SNPs in *LIN28B* with semen quality and reproductive hormones

The analysis of five SNPs in *LIN28B* yielded a genotyping success rate of >99%, and there were no significant deviations from Hardy-Weinberg equilibrium in either the cases or controls (*P* > 0.05). The allele frequency distribution in the control group was similar to the American population from the 1000 Genomes Project Phase 3 (Supplementary Table 3).

We evaluated whether the five SNPs in *LIN28B* confer susceptibility to oligozoospermia using logistic regression analysis in four different comparative models: co-dominant (CoDom), dominant (Dom), recessive (Res) and additive (Add). No statistically significant differences were detected when grouping cases for sperm concentration (<15 × 10^6^/mL) or total sperm count (<39 × 10^6^/mL) in any genetic model. Likewise, no association was observed with sperm concentration, total sperm count and motility as continuous variables. Subsequently, we investigated the possible effect of the five SNPs on FSH, LH and total testosterone levels in all patients and stratified by cases or controls. A positive effect on total testosterone level was observed for the minor allele (A) of rs314277 in all patients (Dom: *P* = 0.032; Add: *P* = 0.024), and for the minor allele (C) of rs7759938 (Dom: *P* = 0.025; Add: *P* = 0.043) and for the minor allele (A) of rs314280 (Dom: *P* = 0.024) in cases (Table 2 and Supplementary Table 4). Total testosterone levels increased 15.2% (*β*_(back transformed)_ = 1.15) and 14.5% (*β*_(back transformed)_ = 1.14) in cases carrying one or two minor alleles of rs7759938 and rs314280 variants, respectively, compared with cases homozygous for the major allele. No associations were observed with FSH and LH levels.

**Table 2:** Significant associations of testosterone levels with *LIN28B* variants in men with oligozoospermia.

	*n*	Mean	SE	Dif	CI (95%)		*P*-value	*P*-value (adjusted by BMI)
**All subjects**								
rs314277_A								
Dominant								
C/C	304	11.13	0.21	*ref*			**0.039**	**0.032**
C/A–A/A	61	12.24	0.52	1.108	0.059	2.157		
Additive								
0, 1, 2				1.101	0.078	2.123	**0.035**	**0.024**
**Cases (oligozoospermia)**								
rs7759938_C								
Dominant								
T/T	55	11.28	0.48	*ref*			**0.031**	**0.025**
T/C–C/C	55	12.97	0.59	1.695	0.201	3.190		
Additive								
0, 1, 2				1.329	0.005	2.654	**0.048**	**0.043**
rs314280_A								
Dominant								
G/G	42	11.09	0.56	*ref*			**0.037**	**0.024**
G/A–A/A	67	12.54	0.46	1.457	0.010	2.904		

The testosterone levels are expressed as nmol/L. Dif.: difference between means. The *P*-values indicate the statistical significance obtained from the analysis conducted with the log-transformed testosterone values. Bold indicates statistical significance, *P* < 0.05.

### The effect of *LIN28B* SNPs on transcript abundance

To determine whether the *LIN28B* SNPs could affect the expression of *LIN28B* and hence act as expression quantitative trait loci (eQTL), a linear regression analysis was performed with the relative abundance level of *LIN28B* transcript. No significant association was observed in all patients jointly, but a stratified analysis revealed a significant association of the minor allele (T) of rs395962 with *LIN28B* mRNA abundance among cases (Dom: mean difference = −0.037, *P* = 0.032; Add: mean difference = −0.026, *P* = 0.057). The presence of at least one minor allele (T) of rs395962 confers a significantly lower abundance of *LIN28B* transcript.

Using publicly available data from GTEx, we verified that rs395962, as well as three of the other studied SNPs, associates with the expression level of *LIN28B* mRNA in testicular tissue of adult men. The analysis shows a decreasing abundance of *LIN28B* mRNA in subjects with heterozygous or homozygous minor genotype in each SNP (*n* = 322, *β* = −0.13, *P* = 0.006 for rs395962_T; *β* = −0.11, *P* = 0.01 for rs7759938_C; *β* = −0.11, *P* = 0.01 for rs314268_G and *β* = −0.12, *P* = 0.004 for rs314280_A) (Supplementary Fig. 6).

## Discussion

In this study, we hypothesized that LIN28B, as a genetic factor that affects the timing of puberty, may also be important for the complete maturation of testicular capacity, regulating the spermatogenic and endocrine function of the testis. Using sperm as an available model of germ cells, we show that *LIN28B* transcript is less abundant in sperm cells from men with idiopathic oligozoospermia, suggesting a downregulation of *LIN28B* expression in adult germ cells. Supporting the above, we revealed that lower *LIN28B* mRNA abundance associates with the minor allele (T) of the *LIN28B* genetic variant rs395962. The rs395962_T variant has been strongly associated with delayed voice breaking in men (6, 8). Even though rs395962 is an intronic variant located in a non-regulatory region, the variant rs314280, in high LD with rs395962 (D´ = 1), is located in a putative regulatory region of 2,000 bp with enhancer activity (Ensemble regulation release 113 – October 2024, accession date 3 March 2025), suggesting a potential role in regulating *LIN28B* transcriptional expression. Thus, LIN28B appears to be mechanistically involved in linking pubertal timing with adult spermatogenic capacity through its expression in the testicular germ cells and potentially in other target tissues such as the hypothalamus, all of which may be modified by the cumulative effect of common genetic variants in the gene sequence.

The LIN28 genes have been extensively studied for their role in regulating cell division, growth and differentiation through their interaction with *let-7* microRNAs. In the present study, we also investigated the expression of two members of the *let-7* microRNA family in spermatozoa of oligozoospermic men. The relative quantification showed an increase in the abundance of *hsa-let-7c-5p* concomitantly with a significantly lower abundance of the *LIN28B* transcript in sperm cells of oligozoospermic men, which is in agreement with the proposed regulation-loop (30). In support of our findings, previous studies in sperm from oligozoospermic men had found upregulation of *let-7c* and other members of the *let-7* family (31, 32). Moreover, interesting studies using *in vitro* models of undifferentiated spermatogonia showed that retinoic acid-induced upregulation of members of the let-7 family is associated with a reduction in cell proliferation and progression to spermatogonia differentiation (33, 34), in contrast to the opposite role of LIN28B in promoting cell proliferation (35). Therefore, the previous evidence and the results of this study suggest that LIN28B may act as a proliferation driver in the most undifferentiated spermatogonia, and that a decrease in its expression could impair spermatogenic capacity, resulting in oligozoospermia in adult men.

On the other hand, some evidence suggests that LIN28B contributes to the regulation of sex hormone pathways and might explain its association with growth and puberty (36). Supporting this assumption, Leinonen *et al.* (36) found a positive correlation of *LIN28B* mRNA expression with the expression of the androgen receptor (*AR*) mRNA in the hypothalamus and pituitary of adult men. Moreover, the authors report the genome-wide association of the *LIN28B* genetic variant rs7759938 with total testosterone in adult men from the UK Biobank database, showing that the pubertal timing-delaying allele C of rs7759938 was associated with lower levels of serum testosterone in healthy men from the general population (36), and subsequently, linking a genetic factor of puberty onset with adult testicular function. In the present study, we observed a positive association of the variants rs7759938_C, rs314280_A and rs314277_A with the level of testosterone. In our patients with idiopathic oligozoospermia, a negative effect of these genetic variants on testosterone levels could be masked by Leydig cell dysfunction and deregulation of the testosterone to LH ratio, characteristic of subjects with spermatogenic failure. Regardless of the direction of the effect, it is remarkable that we also found the association of these polymorphisms with total testosterone levels in oligozoospermic patients, supporting that LIN28B is involved in the regulation of the testosterone pathway in infertile men. It would be interesting to investigate the genes targeted by the LIN28B/let-7 system that finally would regulate these mechanisms. In this sense, some evidence has found a positive correlation between the expression of LIN28B and AR in the hypothalamus, indicating that LIN28B exerts a positive regulatory effect on the expression of *AR* in endocrine tissues (37, 38) and through interaction with *let-7c* (39).

Some limitations can be recognized in our work. First, despite the exclusion of cases with clinical suspicion of obstructive oligozoospermia, it is plausible that some of our cases may have had oligozoospermia of non-secretory origin due to the fact that our selection criteria are not based on testicular histology (40). Second, the sample size for the genetic association study is small, and a greater number of subjects would increase the statistical power of the study. On the other hand, one of the strengths of our mRNA expression analysis is the appropriate number of subjects, which reduces the effect of the intrinsic variability of human samples. Moreover, the specificity of the transcripts to germ cells allowed us to determine with certainty that the quantification of the *LIN28B* transcript is specific to sperm cells in the ejaculate, and given the practically null transcriptional activity of spermatozoa, we speculate that LIN28B transcript abundance in sperm comes from the expression in immature germ cells. Finally, our data concerning the relative age of puberty onset, despite the challenges associated with obtaining this information from adult males, possess the advantage of being collected without bias regarding the seminal quality of the participants. These results corroborate the hypothesis proposed in cohort studies involving younger males, which propose a link between older age at puberty and reduced seminal quality.

Overall, our findings suggest that *LIN28B* is downregulated in germ cells from oligozoospermic patients and that SNPs in *LIN28B*, known to affect pubertal onset, contribute to the lower expression in germ cells. In addition, the association of *LIN28B* genetic variants and total testosterone levels in secretory oligozoospermic men provides a potential mechanistic link between the regulation of pubertal timing and adult testicular function.

## Supplementary materials



## Declaration of interest

The authors declare that there is no conflict of interest that could be perceived as prejudicing the impartiality of the work reported.

## Funding

This work was supported by a grant from the National Fund for Scientific and Technological Developmenthttps://doi.org/10.13039/501100002850 (FONDECYT) of Chile to M.C.L. (grant number 11200898). We would like to express our gratitude to the Programa de Estímulo a la Excelencia Institucional (PEEI) at the University of Chile for assisting with the publication fee.

## Author contribution statement

M.C.L. conceived and designed the study, analysed the data and wrote the manuscript. M.D.F. performed seminal analysis, collaborated in the recruitment of subjects and made critical revision of the manuscript. E.O. recruited the subjects and performed qPCR and IF protocols. G.I. performed hormonal analysis and critical revision of the manuscript. P.I. performed seminal analysis and collaborated in the recruitment of subjects. C.E. performed qPCR protocols and analysed the results. M.E. performed andrological evaluations. K.A. contributed to the conceptualization of the study and made critical revision of the manuscript. A.C. contributed to the conceptualization of the study and made critical revision of the manuscript.
