# Supplementary material for: LIN 28B expression is downregulated in mature spermatozoa of oligozoospermic men and associates with genetic variants previously linked to pubertal onset
Source: Endocr Connect. 2025 May 28;14(6):e250044. doi: 10.1530/EC-25-0044 (PMC12120926; doi:10.1530/EC-25-0044)
Supplement: Supplementary file 1 [file supplementary_materials.pdf]

**Supporting Information**

## Supplementary materials and methods

### RNA and DNA extraction from testicular tissues and blood samples

Testicular tissues were obtained from azoospermic or severe oligozoospermic patients who underwent testicular biopsy for sperm retrieval at our infertility clinic. The patients consented to the use of their samples in an anonymized form (Approval No. 040-2011, Servicio de Salud Metropolitano Central (SSMC) of the Health Ministry of Chile) [1]. A piece of testicular tissue was fixed in Bouin's solution to perform the histological analysis. An additional adjacent piece of testicular tissue was immersed in RNA stabilization solution (RNAlaterTM, Ambion), and stored at −80°C to RNA extraction using a silica-membrane RNA binding column (RNeasy Mini Kit, Qiagen) and DNase digestion (RNase-Free DNAase Set, Qiagen). An aliquot of 1 μg of total RNA was used for complementary DNA synthesis following the manufacturer's instructions (RevertAid H Minus M-MulV, MBI Fermentas).

Peripheral blood samples for the extraction of leucocyte DNA and RNA were obtained from men recruited in this study. Genomic DNA was extracted using the Wizard® genomic DNA purification kit (Promega) following the manufacturer’s instructions. RNA was extracted after purification of peripheral blood mononuclear cells on a gradient of HISTOPAQUE®-1.077 (Sigma-Aldrich) and immediately used for total RNA extraction (RNase-Free DNAase Set, Qiagen).

1. Lardone, M.C., et al., *Histological and hormonal testicular function in oligo/azoospermic infertile men.* Andrologia, 2013. **45**(6): p. 379-85.

**Supplementary Figure 1. Normalization of hsa-miR-30a-5p internal control against unit mass.** The abundance of the hsa-miR-30a-5p transcript (Ct), determined by qRT-PCR, was normalized against the total quantity of RNA employed for the synthesis of complementary DNA (right panel). Extracted RNA was measured with a fluorometric assay (Qubit™ RNA HS Assay Kit) (left panel). Mann–Whitney U test was used to test for statistically significant differences between the groups. A p-value < 0.05 was considered statistically significant.

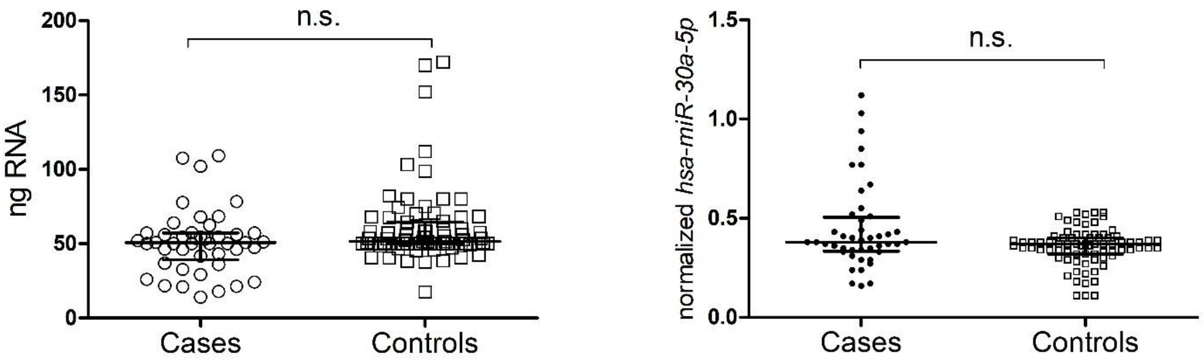

**Supplementary Figure 2. Heatmap matrix of pairwise linkage disequilibrium statistic.** D´ values (blue) and $R^2$ (red) for pairwise comparison in combined European and American population. Data was calculated with the web application LDlink (https://ldlink.nci.nih.gov/?tab=ldmatrix).

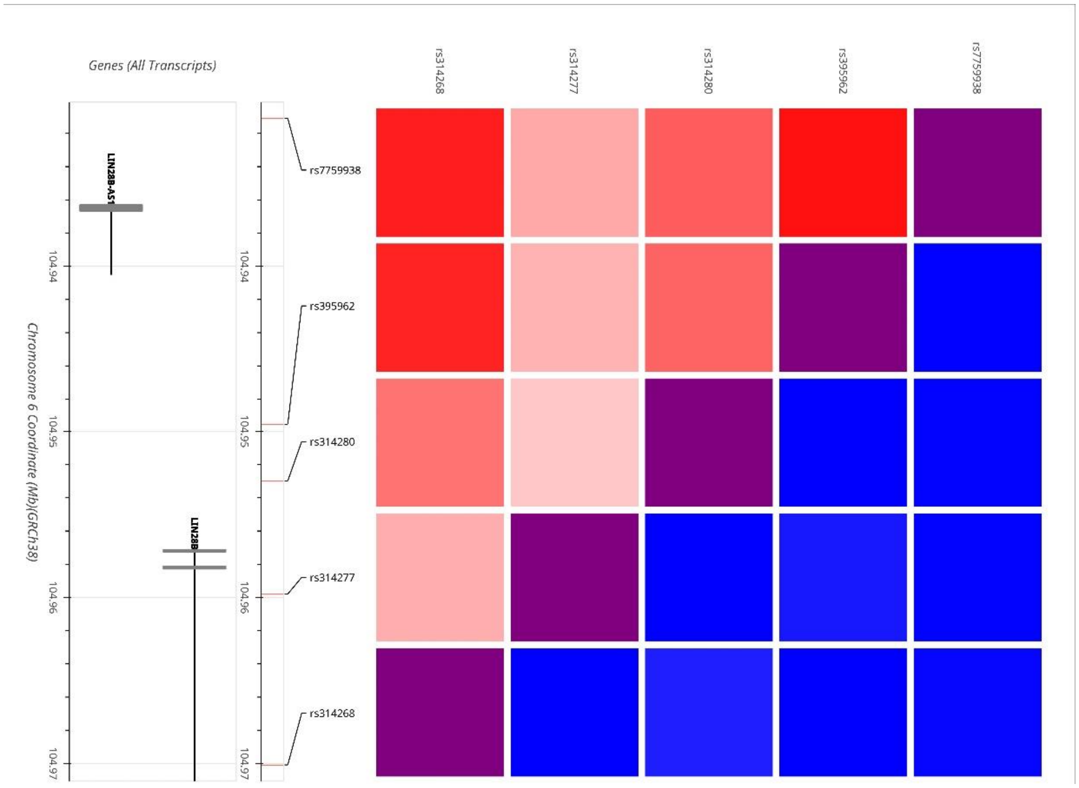


D'

| rs_number | rs7759938 | rs395962 | rs314280 | rs314277 | rs314268 |
|---|---|---|---|---|---|
| **rs7759938** | 1.0 | 0.992 | 1.0 | 0.983 | 0.97 |
| **rs395962** | 0.992 | 1.0 | 1.0 | 0.768 | 1.0 |
| **rs314280** | 1.0 | 1.0 | 1.0 | 1.0 | 0.798 |
| **rs314277** | 0.983 | 0.768 | 1.0 | 1.0 | 1.0 |
| **rs314268** | 0.97 | 1.0 | 0.798 | 1.0 | 1.0 |

$R^2$

| rs_number | rs7759938 | rs395962 | rs314280 | rs314277 | rs314268 |
|---|---|---|---|---|---|
| **rs7759938** | 1.0 | 0.869 | 0.725 | 0.329 | 0.786 |
| **rs395962** | 0.869 | 1.0 | 0.639 | 0.228 | 0.738 |
| **rs314280** | 0.725 | 0.639 | 1.0 | 0.247 | 0.551 |
| **rs314277** | 0.329 | 0.228 | 0.247 | 1.0 | 0.285 |
| **rs314268** | 0.786 | 0.738 | 0.551 | 0.285 | 1.0 |

**Supplementary Figure 3.** Detection of *LIN28B* and *GAPDH-S* transcripts by RT-PCR in RNA extracted from spermatozoa, testicular tissue with normal spermatogenesis, leucocytes, and from DNA of peripheral blood cells.

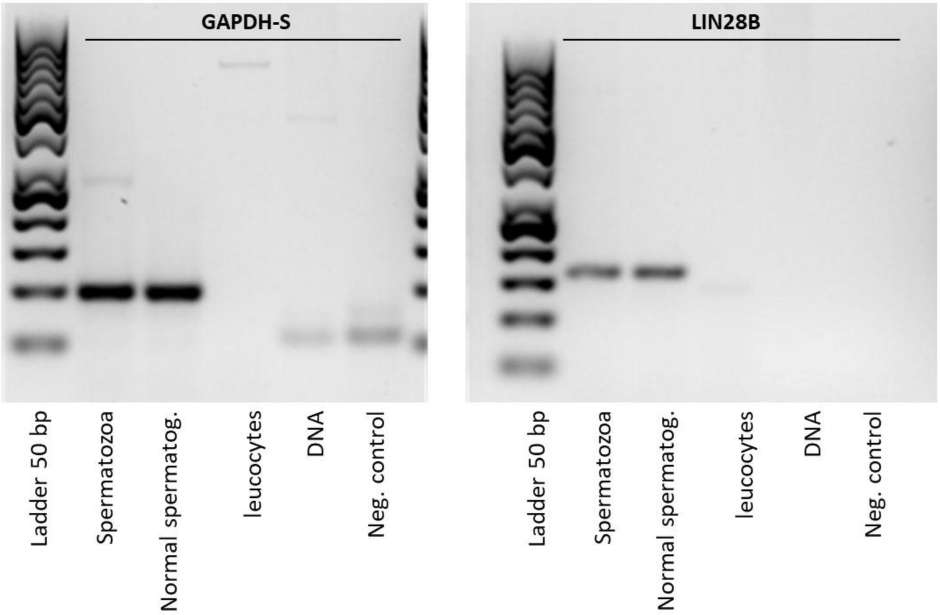


**Supplementary Figure 4. Detection of *LIN28B* and *GAPDH-S* transcripts in RNA extracted from testicular tissues with normal and impaired spermatogenesis by RT-PCR.** SCOS: Sertoli cell only syndrome. GAPDH: glyceraldehyde-3-phosphate dehydrogenase, ubiquitous expression. GAPDH-S: glyceraldehyde-3-phosphate dehydrogenase spermatogenic, sperm-specific enzyme.

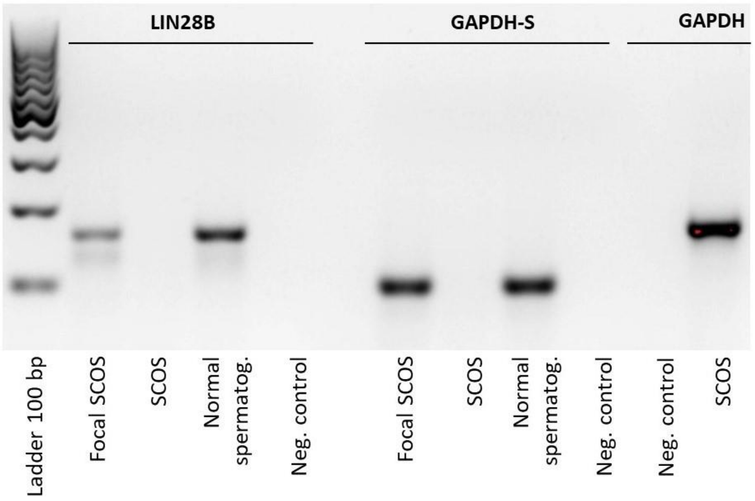

**Supplementary Figure 5. Correlation between *LIN28B* normalized expression and *let-7c* normalized expression in mature sperm cells of cases and controls.** Spearman correlation test. The lines show the regression line and the 95% confidence band (dotted lines).

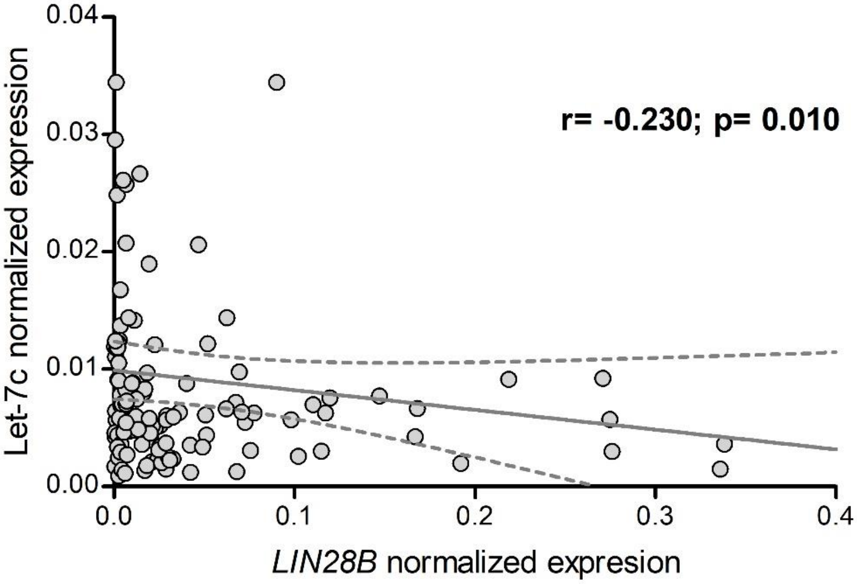

**Supplementary Figure 6. Violin plot of normalized expression of *LIN28B* transcript in the three genotypes for five SNPs.** Data extracted from GTEx project version 8, date 04-06-2024). P values of regression analysis are shown at the bottom (β= -0.13, p=0.006 for rs395962_T; β= -0.11, p=0.01 for rs7759938_C; β= -0.11, p=0.01 for rs314268_G and β= -0.12, p=0.004 for rs314280_A). The beta coefficient is the normalized effect size, and its magnitude has no direct biological interpretation. In parenthesis is shown the number of testicular tissue samples with transcript expression quantification and genotyped.

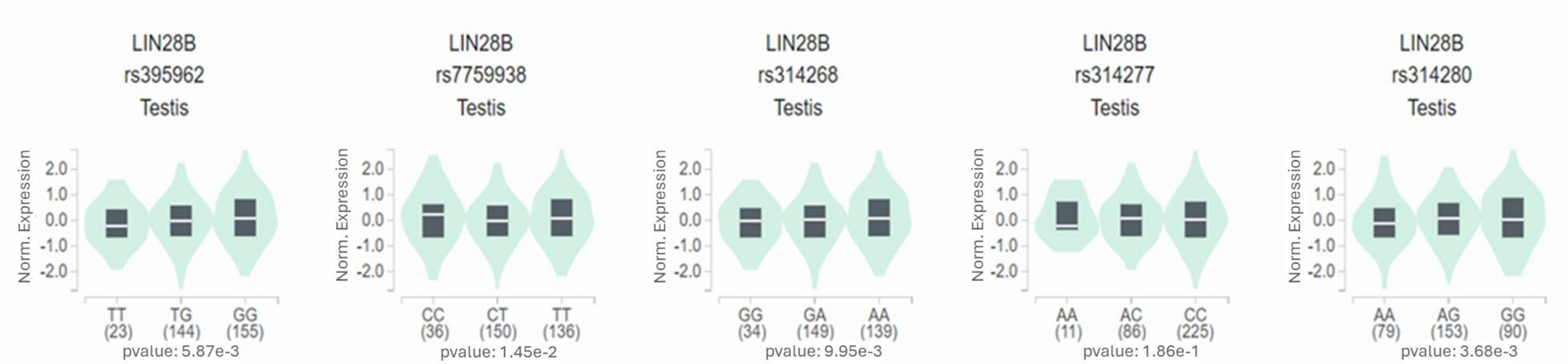

**Supplementary Table 1. Semen quality of patients who consulted for infertility according to self-reported onset of puberty**

| Relative age of puberty | N | Sperm Concentration (million/ml) | Total Sperm Count (million) | Progressive motility (%) | Normal morphology (%) |
|---|---|---|---|---|---|
| Early | 22 | 57 (29-108) **a** | 127 (72-220) **a** | 70 (53-76) **a,b** | 3 (1-5) **a,b** |
| Synchronic | 239 | 48 (20-86) **b** | 119 (42-255) **b** | 61 (43-71) **b** | 2 (1-3) **b** |
| Late | 59 | 24 (12-60) **a,b** | 63 (16-169) **a, b** | 51 (33-65) **a,b** | 1 (0-3) **a,b** |

Values are expressed as median (25th-75th percentile) Same letter indicates the groups with statistically significant difference between them, $p<0.05$, Mann-Whitney *U* test

**Supplementary Table 2. Percentage of change in semen parameters and FSH levels in patients who consulted for infertility according to self-reported onset of puberty.**

| Relative age of puberty | N | Sperm Concentration[a] (%, 95% CI) | Total Sperm Count[a] (%, 95% CI) | FSH[a] (%, 95% CI) |
|---|---|---|---|---|
| *Model I* | | | | |
| Early | 22 | 12.5, -26.9; 73.2 | 19.8, -24.6; 90.4 | -5.8, -22; 13.7 |
| Synchronic | 239 | reference | reference | reference |
| Late | 59 | -22.9, -41.5; 1.7 | -25.1, -44.3; 0.9 | 12.3, -0.9; 27.2 |
| *Model II* | | | | |
| Early | 22 | 16.9, -24.9; 82 | 23.8, -23.1; 99.2 | -6.3, -22.8; 13.7 |
| Synchronic | 239 | reference | reference | reference |
| Late | 59 | -22.8, -41.5; 1.9 | -24.9, -44.2; 1.2 | 12.1, -1.1; 27.1 |

Model I: Sperm concentration and Total sperm count adjusted for days of abstinence and age. FSH adjusted for time of blood sampling and age. Model II: Sperm concentration and Total sperm count adjusted for days of abstinence, age and BMI. FSH adjusted for time of blood sampling, age and BMI.
[a] Transformed by the use of natural logarithm and back transformed giving the percentage change.

**Supplementary Table 3. Minor allele frequency (MAF) of selected single nucleotide polymorphisms (SNPs) in this study and in different populations according to 1000 Genomes Project Phase 3[1].**

| SNP | This study (controls) | This study (cases) | European population[1] | American population[1] |
|---|---|---|---|---|
| **rs7759938_C** | 0.25 | 0.27 | 0.35 | 0.28 |
| **rs395962_T** | 0.24 | 0.24 | 0.34 | 0.26 |
| **rs314268_G** | 0.26 | 0.26 | 0.35 | 0.32 |
| **rs314277_A** | 0.08 | 0.09 | 0.16 | 0.12 |
| **rs314280_A** | 0.36 | 0.37 | 0.47 | 0.35 |

[1] **1000 Genomes Project Phase 3.** Revised for every SNP in March 2025.

**Supplementary Table 4. P-values for the association analysis of testosterone levels with *LIN28B* SNPs in men with oligozoospermia and in all subjects.**

| **SNPs** | *Genetic model* | | | |
|---|---|---|---|---|
| | Codominant | Dominant | Recessive | Aadditive |
| | | *Cases (oligozoospermia)* | | |
| **rs7759938** | 0.099 (0.081) | **0.031 (0.025)** | 0.813 (0.830) | **0.048 (0.043)** |
| **rs395962** | 0.418 (0.341) | 0.185 (0.142) | 0.813 (0.830) | 0.216 (0.179) |
| **rs314268** | 0.290 (0.301) | 0.117 (0.121) | 0.588 (0.830) | 0.125 (0.156) |
| **rs314277** | 0.173 (0.053) | | | |
| **rs314280** | 0.116 (0.066) | **0.037 (0.024)** | 0.580 (0.922) | 0.074 (0.098) |
| | | *All subjects* | | |
| **rs7759938** | 0.603 (0.496) | 0.842 (0.760) | 0.315 (0.236) | 0.595 (0.490) |
| **rs395962** | 0.794 (0.785) | 0.730 (0.812) | 0.627 (0.564) | 0.901 (0.996) |
| **rs314268** | 0.293 (0.406) | 0.933 (0.956) | 0.135 (0.189) | 0.619 (0.588) |
| **rs314277** | 0.104 (0.062) | **0.039 (0.032)** | 0.444 (0.209) | **0.034 (0.024)** |
| **rs314280** | 0.201 (0.209) | 0.313 (0.317) | 0.084 (0.088) | 0.112 (0.115) |

The P-values indicate the statistical significance obtained from the analysis conducted with the log-transformed testosterone values. In parentheses is shown the p-value after adjustment by BMI
